# Central Nervous System Complications in Cystinosis: The Role of Neuroimaging

**DOI:** 10.3390/cells11040682

**Published:** 2022-02-15

**Authors:** Aude Servais, Jennifer Boisgontier, Ana Saitovitch, Aurélie Hummel, Nathalie Boddaert

**Affiliations:** 1 Department of Nephrology and Transplantation, Centre de référence des Maladies Rénales Héréditaires de l’Enfant et de l’Adulte, Necker Hospital, AP-HP, 75015 Paris, France; aurelie.hummel@aphp.fr; 2 INSERM U1163, Imagine Institute, Paris University, 75015 Paris, France; boisgontier.jennifer1414@gmail.com (J.B.); a.saitovitch@gmail.com (A.S.); nathalie.boddaert@aphp.fr (N.B.); 3 Paediatric Radiology Department, INSERM U1299, Hôpital Necker Enfants Malades, AP-HP, 75015 Paris, France

**Keywords:** cystinosis, central nervous system, cortical atrophy, arterial spin labelling, cysteamine, cystine blood level

## Abstract

Despite improvement in the specific treatment, clinical and anatomo-functional central nervous system (CNS) abnormalities of various severities are still observed in cystinosis patients. Patients who develop CNS complications today have a worse compliance to cysteamine treatment. Radiological studies have shown that cortical or central (ventriculomegaly) atrophy is observed in more than two thirds of cystinosis patients’ magnetic resonance imaging (MRI) and correlates with the intelligence quotient score. Half of cystinosis patients have marked aspecific white matter hyperintensities. The development of advanced neuroimaging techniques provides new tools to further investigate CNS complications. A recent neuroimaging study using a voxel-based morphometry approach showed that cystinosis patients present a decreased grey matter volume in the left middle frontal gyrus. Diffusion tensor imaging studies have shown white matter microstructure abnormalities in children and adults with cystinosis, respectively in areas of the dorsal visual pathway and within the corpus callosum’s body. Finally, leucocyte cystine levels are associated with decreased resting cerebral blood flow, measured by arterial spin labelling, in the frontal cortex, which could be associated with the neurocognitive deficits described in these patients. These results reinforce the relevance of neuroimaging studies to further understand the mechanisms that underline CNS impairments.

## 1. Introduction

Cystinosis is a rare autosomal recessive disease caused by intracellular cystine accumulation [1,2]. Three clinical forms have been described based on the severity of symptoms and the age of onset: infantile cystinosis characterized by renal proximal tubulopathy and early progression to end-stage renal disease (ESRD), a juvenile form with a markedly slower rate of progression and an adult form with mainly, but not only, ocular abnormalities [3]. Over the last 20 years, specific treatment with cysteamine and progress in renal transplantation and dialysis have significantly improved the long-term outcome of cystinosis patients. However, extra-renal complications may still occur [4]. In particular, in historical cohorts, central nervous system complications (CNS) were observed in adolescents and young adults not fully treated with cysteamine, as this treatment became available only in the 1990s [5,6,7]. Today, in the era of early specific treatment with cysteamine, a high prevalence of mild to severe clinical and radiological CNS impairments is still observed in adult patients. Such complications may affect quality of life, academic function, and professional insertion. The development and availability of advanced neuroimaging techniques provides new tools to investigate the underlying neurophysiopathological mechanisms of metabolic diseases and further understand their impact on broader neurological and neurocognitive dysfunction [8]. However, studies in this domain are still scarce, and only two have specifically used such an approach.

## 2. Clinical Presentation

### 2.1. Clinical Data

Neurological symptoms have been reported in adolescents and young adults with cystinosis in historical cohorts [5,6,7]. Two main clinical forms have been observed. The first one is a cystinosis encephalopathy with cerebellar signs and/or motor difficulties, mainly of the lower limbs, a decrease of oral expression, and the progressive development of pyramidal symptoms, somnolence, epileptic seizures, and mental deterioration [7,9]. Motor coordination difficulties were initially described and have been further corroborated in more recent studies of motor performance [10,11,12]. The second form resembles a stroke-like episode, which could present itself with coma or hemiplegia or milder symptoms. In parallel, hydrocephalus has also been described in some patients [13], not necessarily associated with clinical symptoms [7]. Cerebrospinal fluid examination is normal except for elevated pressure, suggesting that the hydrocephalus might result from decreased cerebrospinal fluid absorption related to the deposition of cystine in choroidal plexuses and meninges [13,14].

CNS complications are a major concern since the long-term prognosis of adult cystinosis patients appears to be primarily related to neurological complications [15]. Indeed, in a French cohort, neurological disorders were globally reported in 37% of patients and included paresis in 75%, cognitive impairment in 56%, stroke in 37%, and seizures in 31% [15]. In addition, the cause of death was linked to neurological reason in one-third of cases in that series and in two patients out of thirty-three (6%) in the series of Gahl et al. [16]. These data further reinforce the relevance of investigating CNS complications in this disease. A more recent study, specifically focusing on CNS complications, included 18 adults with infantile cystinosis [17]. In that cohort, CNS complications were largely present, despite early diagnosis and treatment in most patients. Seven patients (39%) presented with at least one central nervous system clinical abnormality: 2 (11%) with seizures, 3 (17%) with memory impairment, and 5 (28%) with cognitive impairment. Of note, patients with renal diseases and in particular cystinosis patients may have electrolyte abnormalities that can also trigger seizures, without implying a central nervous system etiology. One patient (5%) presented with a stroke-like episode. Mini-mental state examination, which screens for cognitive function deficit, was assessed in twelve patients, and the median score was twenty-seven out of thirty, with four patients having a decreased score below twenty-five.

### 2.2. Risk Factors for the Development of Central Nervous System Complications

In historical publications, CNS complications’ occurrence did not correlate with other extra-renal complications of cystinosis, but their frequency was directly correlated with age in the absence of early treatment [5,6,7]. Indeed, the impact of specific treatment on such complications has been described [7,15,16,18]. Interestingly, it has been demonstrated that cysteamine intake could decrease neurocognitive impairments in neurodegenerative diseases, such as Huntington’s disease, supporting its action on neurological symptoms, even if the underlying mechanism may be different [19]. A more recent study has shown that patients who develop CNS complication today are those who have a lower compliance score and/or receive a lower cysteamine dose, without an effect of age [17]. In addition, even if other factors such as psychosocial struggle should be considered, all patients with at least one central nervous system symptom had poor compliance to treatment, further reinforcing the role of cysteamine treatment in preventing neurocognitive symptoms. Unfortunately, despite these results, compliance to treatment remains a major challenge in the context of cystinosis, in particular in adults [20]. The effect of treatment on already-existing neurological complications is less clear, but the early identification of such neurological symptoms could help in the adjustment of treatment and prompt referral to a specialized neurologist [7].

## 3. Pathophysiology

The underlying mechanisms of CNS complication remain poorly understood. Investigations using animal models have shown that cystinosin knockout mice, C57BL/6 *Ctns* ^−/−^ mice, have elevated cystine levels in the hippocampus, cerebellum, forebrain, and brainstem, which increase with age. In addition, cystine crystals have been detected within the choroid plexus and situated adjacent to capillaries. This has been associated with spatial reference and working memory deficits [21]. These results support the hypothesis that cystinosis-associated central nervous system anomalies are due, at least in part, to progressive cystine accumulation.

**Figure 1:**
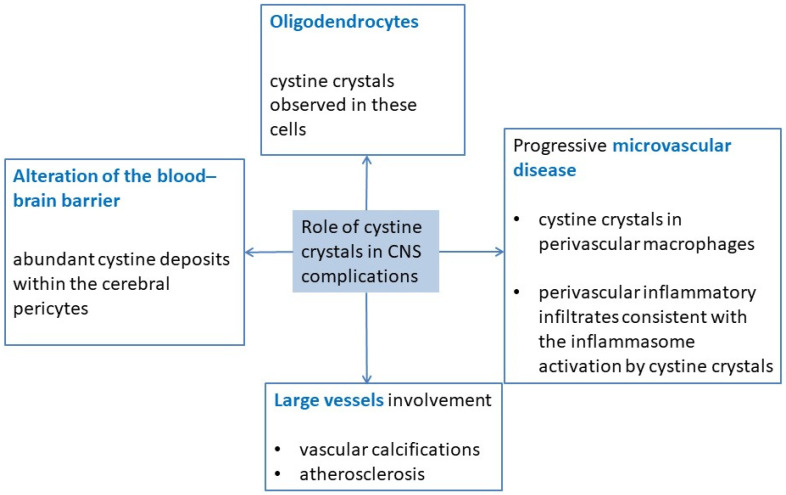
Role of cystine crystals in CNS complications: pathophysiological hypotheses.

The exact pathogenic role of cystine crystals remains unknown. Several non-exclusive hypotheses have been formulated (Figure 1). Firstly, it could involve oligodendrocytes since cystine crystals have been observed in these cells [7,22,23]. A second hypothesis is the alteration of the blood–brain barrier based on the finding of abundant cystine deposits within the cerebral pericytes, which contribute to this barrier [22]. A third hypothesis is the progressive development of a microvascular disease of the brain. Indeed, cystine crystals have been observed in perivascular macrophages [7], and a cerebral cystine-crystal-associated vasculopathy with perivascular inflammatory infiltrates has been described [22,24], consistent with the inflammasome activation by cystine crystals already shown [25]. In addition to microvascular lesions, large vessel involvement has been reported, and this may be the cause of other types of cerebral complications [26,27,28]. Accumulation of intracellular cystine itself may be a risk factor for vascular calcifications, even if adult cystinotic patients usually have several other risk factors for vascular calcification and atherosclerosis since most patients have endured renal failure and have undergone at least one renal allograft procedure [18,29].

## 4. Neurocognitive Impairments

The investigation of neurocognitive impairment in cystinosis patients has shown that even if they generally do not have intellectual disability, mild neurocognitive impairment can be present [10,12,30]. Indeed, specific impairments in the processing of visual information, as well as relative weakness in visual motor, visual spatial, and visual memory skills have been described, which may be associated with learning difficulties, primarily in arithmetic. Significant difficulties are observed in executive-function-related abilities [12,31,32,33,34,35]. A fine-motor coordination deficit in children and adolescents with cystinosis has also been documented [11]. In addition, abnormalities seem to increase with age, which may reflect a progressive cognitive impairment, possibly as a result of cystine accumulation in the brain over time [30]. Impaired working memory has also been described, with visual memory being more impaired than auditory memory in one study [35,36].

Interestingly, there is a significant correlation between the total intelligence quotient (IQ) and the age at cysteamine treatment start: the sooner cysteamine is started, the less the IQ is impacted [35]. Of note, all cystinosis patients from the mentioned study who started cysteamine before 2 y of age had an IQ within the normal range, which is in accordance with another study showing that cystinosis patients treated before 2 y of age had a better outcome [12].

## 5. Radiological Data

In adult cystinosis patients, there is a very high prevalence of abnormalities on clinical brain scans: 89% of patients have an abnormal exam using advanced imaging techniques [17].

### 5.1. Calcifications

In historical cohorts, mineralization of the basal ganglia seemed to be specific to severe encephalopathy. In some patients with profound neurological deficits, brain imaging or post-mortem examination revealed multifocal cystic necrosis, dystrophic calcifications of the basal ganglia and periventricular areas, extensive demyelination of the internal capsule, spongy changes in the brachium pontis, and vacuolization [22,37,38]. Cerebral calcifications were observed in 22–38% of cases, but in a more recent study, computed tomography scan found brain calcification in only one patient out of twenty-one who presented with a stroke-like episode [15,16,17].

### 5.2. Cortical Atrophy

**Figure 2:**
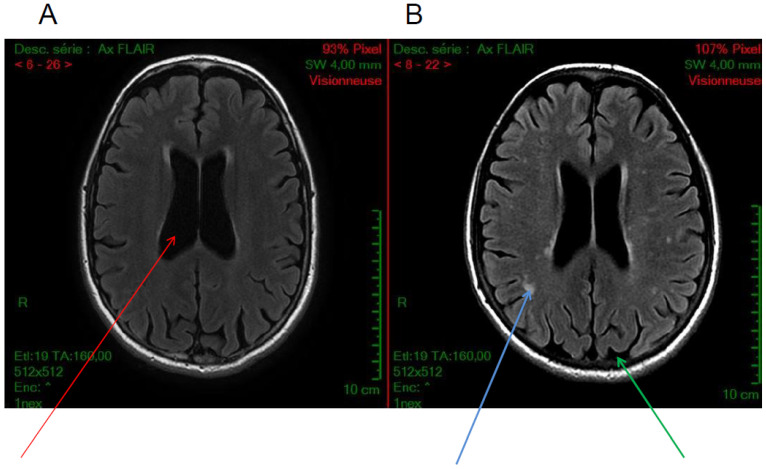

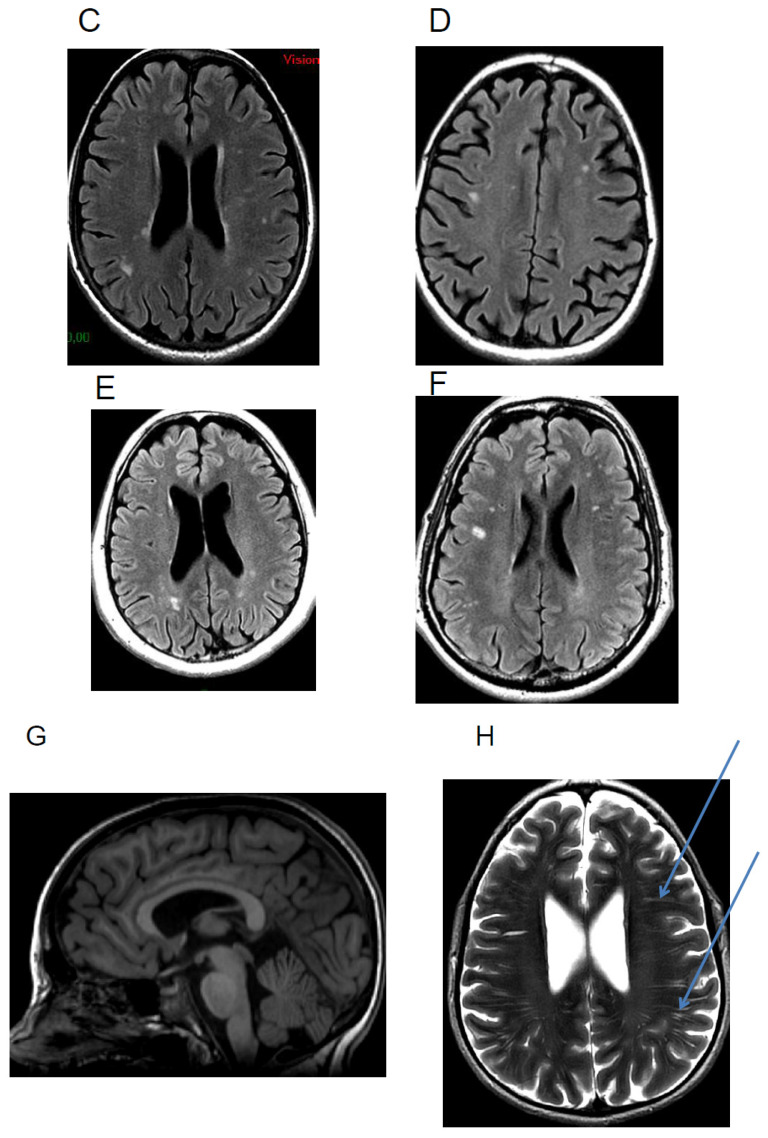
Axial FLAIR sequences. Examples of brain MRI anatomical images of cystinosis patients showing (**A**) an isolated ventricular dilatation (red arrow), (**B**) in another patient, cortical atrophy (green arrow) and diffuse white matter anomalies (blue arrow) associated with ventricular dilatation, (**C**–**F**) diffuse subcortical white matter hyperintensities, and (**G**,**H**) Wirshow or perivascular space enlargement (blue arrow)(images from N. Boddaert, Necker hospital).

Cortical atrophy is the most frequent radiological finding in cystinosis patients. By computed tomography (CT) scan or magnetic resonance imaging (MRI), cortical atrophy is observed in almost all patients with CNS symptoms [7]. Cerebral atrophy is also reported in patients without important CNS clinical abnormality and in patients with minor alterations in cognitive performance, in particular with impairment of visual memory [15,36,39]. In a recent study, among patients with infantile cystinosis, 72% showed evidence of cortical atrophy, 67% central atrophy (ventriculomegaly), and 50.0% demonstrated both (Figure 2) [17]. In that study, only two patients with infantile cystinosis had a normal brain MRI, both being the youngest patients included. Interestingly, no atrophy was observed in patients with late-onset cystinosis, even if the patients analyzed were older than the other cystinosis patients. Importantly, such atrophy was specifically observed in cystinosis patients, and not in controls with nephropathy. In another study, also including younger patients, cystinosis patients presented significantly more atrophy than age- and sex-matched healthy controls in the frontal, parietal, temporal, and occipital regions, the corpus callosum, and the cerebellum [35]. It is worth noticing that atrophy was localized in parieto-occipital regions, which is consistent with the visuo-spatial-specific impairment described in these patients.

In a study conducted with children and adolescents with cystinosis, Trauner et al. investigated brain volume loss and the correlation with motor coordination deficits. The results showed no significant differences in motor coordination scores between the group of patients who presented brain volume loss and the group who did not [11]. In a recent study, Curie et al. found a significant correlation between the degree of brain atrophy and the total IQ score [35]. Indeed, non-atrophic cystinosis patients had a significantly higher IQ than atrophic patients, which confirmed the previous findings [36].

### 5.3. White Matter Hyperintensities

White matter hyperintensities can be observed in 50% of cystinosis patients, including in patients with adolescent onset cystinosis (Figure 2) [17,35]. However, we have recently shown that these hyperintensities can also be observed in other patients with chronic renal failure [17]. White matter anomalies have been reported in some previous studies [7,40]. Interestingly, white matter abnormalities have been described in adults with chronic kidney disease compared to controls, suggesting that chronic kidney disease may result in a brain phenotype consistent with accelerated aging [35,41,42]. These results demonstrate the importance of adding a control group with renal failure when investigating brain abnormalities in cystinosis patients.

### 5.4. Others

It has been described that children with cystinosis have a 12-fold higher prevalence of Chiari I malformations than the general pediatric population. Indeed, Chiari malformation is observed in 9.5% to 18% of patients [17,43,44]. Even though there are usually no clinical manifestations, some patients may present with symptoms or signs thought to be related to the malformation. Surgical decompression is rarely needed [44]. 

Cazals et al. described a case of an adult patient with perivascular uptake of contrast associated with micronodular T2 hypointensity [45], which could represent microhemorragic lesions secondary to small vessel damage. In such situations, susceptibility weighted (SWI) or gradient echo (GRE) imaging would be useful to add to MRI scans to further evaluate the presence of microhemorrhages. Finally, magnetic resonance spectroscopy sequences do not show any cystine peak or any other abnormal peak in patients with cystinosis [17].

## 6. Neuroimaging Investigations of Anatomo-Functional CNS Abnormalities

The development of advanced neuroimaging techniques has provided new tools to study the underlying neurophysiopathological mechanisms of metabolic diseases. Indeed, different MRI sequences allow noninvasively measuring anatomical and functional brain parameters. In addition, computational statistical software allows comparing these parameters between patients and controls, for instance, as well as to investigate putative correlations with clinical profiles or treatments, providing valuable knowledge in the field. However, so far, very few studies have used this approach to investigate CNS abnormalities in cystinosis patients.

### 6.1. Grey Matter

In the only study investigating grey matter abnormalities, we compared anatomical images from cystinosis patients to those from controls with nephropathy and from healthy controls [17]. We used a voxel-based morphometry (VBM) analysis to compare grey matter volume between groups in each and every voxel of the brain. This whole-brain approach allows investigating putative differences in grey matter without an a priori hypothesis. The results showed significantly decreased grey matter in the left middle frontal gyrus in cystinosis patients compared to healthy controls. Interestingly, brain abnormalities within this region could be associated with executive function deficits clinically described in these patients [10,12]. A significant decrease in grey matter in the same region was also observed in controls with nephropathy compared to healthy controls. No significant difference was observed between cystinosis patients and controls with nephropathy, which suggests that these abnormalities may not be specific to cystinosis patients and encourage brain imaging and neurocognitive investigations in a broader range of renal diseases.

### 6.2. White Matter

Beyond a radiological description of white matter abnormalities characterized by hyperintensities in MRI scans, which are largely described in cystinosis patients as presented earlier, the white matter microstructure can also be studied using diffusion tensor imaging MRI sequences. Diffusion tensor imaging measures the random motion, or diffusion, of water molecules in neural tissue and allows inferring the structural characteristics of the local tissue environment underlying the movement, i.e., the white matter itself. Two main indices have been investigated: the mean diffusivity (MD), a measurement of the overall magnitude of diffusional motion, and the fractional anisotropy (FA), as an index of the brain white matter microarchitecture [46]. 

**Figure 3:**
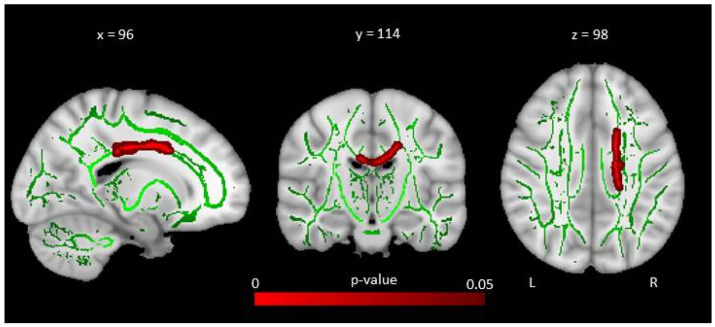
Results from tract-based spatial statistics analyses: voxelwise group differences in fractional anisotropy when comparing cystinosis patients and healthy controls. Sagittal, coronal, and axial slices of the tract-based spatial statistics contrasts between cystinosis patients and healthy controls. Red clusters indicate reduced fractional anisotropy in cystinosis patients compared to healthy individuals. Images of contrast are overlaid on a standard Montreal Neurological Institute (MNI) template 1 mm brain and a fractional anisotropy skeleton (in green) with a threshold set to range from 0.2 to 0.8. Tract-based spatial statistics results are thresholded at *p*  ≤  0.05, corrected for multiple comparisons across space (FWE) using threshold-free cluster enhancement adjusted for age and sex.

In 2010, Bava et al. studied the cerebral white matter microstructure in 24 young children with cystinosis (age 3–7 years) and examined fractional anisotropy and mean diffusivity [43]. Children with cystinosis evidenced a decrease in fractional anisotropy and a corresponding elevation in mean diffusivity, indicating lower fiber integrity and therefore abnormal anatomical connectivity, in areas of the dorsal visual pathway. This suggests that abnormalities in cerebral white matter are present early on in development [43]. Older cystinosis children (>5 years) demonstrated stronger associations between cystine level and mean diffusivity in bilateral parietal regions, suggesting that, in addition to an early disruption in white matter maturation, there might be a secondary progressive effect of cystine accumulation on white matter organization and connectivity [43]. Recently, using tract-based spatial statistics analysis, we investigated white matter microstructure in adults with cystinosis [17]. Our results showed a significantly decreased fractional anisotropy in cystinosis patients compared to healthy controls in clusters within the corpus callosum’s body, indicating a white matter microarchitecture abnormality, which suggests abnormal anatomical connectivity in this region (Figure 3). This bundle, which plays a central role in inter-hemispheric communication, has also recently been associated with cognitive processes [47,48]. However, controls with nephropathy also present with these abnormalities compared to healthy controls, suggesting that they may not be specific to cystinosis patients.

### 6.3. Resting Brain Function

**Figure 4:**
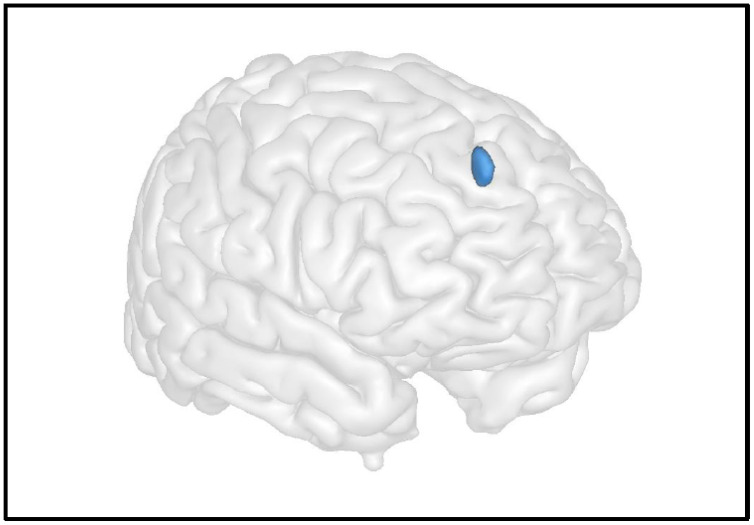
Advanced neuroimaging techniques can help in understanding the impact of cystinosis on the brain’s anatomo-function and its link with neurocognitive impairments. For instance, here, we illustrate the results of Scheme 10 (y = 50 z = 40), a brain region strongly implicated in cognitive functions: the higher the cystine levels, the lower the resting CBF in this area, which is associated with cognitive functions. Maximum intensity projections of T statistics clusters that are significantly correlated with individual cystine blood level are superimposed on a 3D volume rendering on grey matter in the MNI space.

The study of resting brain function provides information on the level of activity of the different brain regions as a “baseline”, outside task performance. Using arterial spin labelling MRI (ASL-MRI), cerebral blood flow (CBF) can be measured at the cerebral level using intrinsic physiological contrast by labelling water protons from cervical arteries and measuring them once they are at the cerebral level [49]. In a recent study, we compared arterial spin labelling images between patients with cystinosis and healthy controls, using a whole-brain approach [17]. We did not find any significant differences in resting cerebral blood flow values between groups. However, in cystinosis patients, the results showed a significant negative correlation between the cystine blood level and resting cerebral blood flow in the right superior frontal gyrus (Figure 4). Indeed, patients with higher levels of cystine were those presenting with lower resting cerebral blood flow values in the superior frontal cortex, which reinforces the link between cystinosis disease and abnormalities within frontal brain regions. Importantly, the superior frontal cortex is associated with executive functions, and the described abnormalities could underline the neurocognitive deficits described in cystinosis patients, such as memory impairments or further cognitive impairments.

## 7. Electrophysiological Activity 

Functional brain activity in cystinosis may be assessed by a high-density electroencephalogram (EEG) [50]. This non-invasive method provides information at the millisecond scale, measures functional brain activity, and thus, assesses the integrity of neural function. A case report tested visual processing in two children with cystinosis before and after kidney transplantation. Before transplantation (and during dialysis), both children showed delayed and decreased early visually evoked responses, compared to their age-matched peers, but with both amplitude and latency measures normalized two years after transplantation [51]. 

High-density EEG was used to analyze basic sensory processing in cystinosis, focusing on early auditory sensory processing (N1) and sensory memory (mismatch negativity) [50]. The auditory sensory processing is the first prominent negative auditorily evoked potential [52] and reflects neural activity generated in and around the primary auditory cortex [53]. The memory mismatch negativity, operating at the sensory memory level, occurs when a repeating stimulus (the standard) in an auditory stream is replaced by a deviant stimulus.

No anomalies have been found in the auditory sensory processing, suggesting that sensory transmission through the auditory system is largely intact in individuals with cystinosis. However, individuals with cystinosis present reduced responses for the longer stimulus onset asynchronies, which could indicate a reduced duration of auditory sensory memory traces, and thus sensory memory impairment, in children and adolescents diagnosed with cystinosis [50]. Adults with cystinosis produce highly similar sensory perceptual auditorily evoked potential responses to controls, suggesting intact early auditory cortical processing. However, significantly increased auditory sensory perceptual processing amplitudes, increased attentional orienting, and reduced sensory memory at slower stimulation rates are observed, suggesting mild-to-moderate changes in auditory sensory memory and attentional processing [54].

## 8. Conclusions

Despite an improvement in specific treatment and transplantation, CNS abnormalities of various severities are still present in adult cystinosis patients. In particular, neurocognitive impairment has been largely described. Unraveling the brain mechanisms that underline these abnormalities is essential to develop actions to improve long-term patient outcome. By MRI, cortical or central atrophy is observed in more than two-thirds of cystinosis patients and correlates with the total IQ score. The development of advanced neuroimaging techniques provides new tools to investigate CNS complications and the brain correlates of neurocognitive impairments. Therefore, future studies, in particular focusing on brain–behavior correlations, can bring new light to the subject. It is important to note that some findings may not be specific to cystinosis, supporting not only the importance of adding a control group with renal failure when investigating brain abnormalities in cystinosis patients, but also the relevance of neuroimaging investigations in patients with renal failure in general. It was recently shown that leucocyte cystine levels are associated with decreased resting cerebral blood flow in the frontal cortex, which could be related to the neurocognitive deficits described in cystinosis patients. These results further reinforce the importance of compliance to cysteamine treatment, which is a major concern in these adult patients, since it seems to play a major role in cognitive and neurological complications.
